# Trends in Oropharyngeal Cancer Incidence Among Adult Men and Women in the United States From 2001 to 2018

**DOI:** 10.3389/fonc.2022.926555

**Published:** 2022-07-18

**Authors:** Fangjian Guo, Mihyun Chang, Matthew Scholl, Brian McKinnon, Abbey B. Berenson

**Affiliations:** ^1^ Center for Interdisciplinary Research in Women’s Health, The University of Texas Medical Branch, Galveston, TX, United States; ^2^ Department of Obstetrics and Gynecology, The University of Texas Medical Branch, Galveston, TX, United States; ^3^ Department of Otolaryngology-Head and Neck Surgery, The University of Texas Medical Branch, Galveston, TX, United States

**Keywords:** oral squamous cell carcinoma, oral cancer, epidemiology, epidemiology and prevention, human papillomavirus

## Abstract

**Background:**

The human papillomavirus (HPV) vaccine was approved in 2006 and has been shown to decrease vaccine-related HPV types in the oropharynx. Its impact on the incidence of HPV-related oropharyngeal squamous cell carcinoma (OPSCC) has not been examined. We investigated the impact of HPV vaccination on the incidence of HPV-related OPSCC in the US among male and female adults from different age groups.

**Methods:**

The US Cancer Statistics 2001–2018 database and the National Cancer Institute (NCI)’s Surveillance Epidemiology and End Results (SEER) program were used in this study. OPSCC incidence was age-adjusted to the US standard population in 2000. Cause-specific 5-year survival probability was calculated using 60 monthly intervals in SEER*Stat software.

**Results:**

Incidence of HPV-related OPSCC was much higher in males than in females. Age-adjusted annual incidence of OPSCC was significantly lower in 2014-2018 than in 2002-2006 among males 20-44 years old (11.4 vs 12.8 per 1,000,000, rate ratio 0.89, 95% confidence interval 0.84-0.93) and among females 20-44 years old (3.0 vs 3.6 per 1,000,000, rate ratio 0.86, 95% confidence interval 0.78-0.95), but increased in both 45-64 year old and 65+ year old males and females. Joinpoint regression revealed a significant joint in the HPV-OPSCC incidence trend for 20-44-year-old males in 2008 at which time the incidence began to decrease. Except for 20-44 year old females (74.8% in 2002-2006 vs. 75.7% in 2009-2013, p=0.84), cancer-specific 5-year survivals significantly improved for males and females of all age groups.

**Conclusions:**

HPV-related OPSCC was much more common in males. Incidence of HPV-related OPSCC declined among young adults during the vaccination era compared with pre-vaccination era. Cancer-specific 5-year survival was significantly improved in young males but not in young females.

## Introduction

A persistent oral human papillomavirus (HPV) infection places a person at risk of developing oropharyngeal squamous cell carcinoma (OPSCC) (1–3). OPSCC patients whose cancer tests positive for HPV fare better than those whose cancer tests negative for the virus (3–6). Previous reports revealed increasing incidence of HPV-related OPSCC in both middle-aged and elderly adult populations in the United States (US) (7, 8). The incidence of HPV-related oral squamous cell carcinoma was reported to steadily increase from 1973 to 2004, whereas HPV-unrelated oral squamous cell carcinomas did not increase during this time (7). In elderly patients ≥65 years of age, the incidence of OPSCC was also reported to increase from 2000 to 2012, mainly due to the increasing incidence of HPV-related OPSCC (8).

Prior estimates of OPSCC incidence in the US were largely based on the National Cancer Institute (NCI)’s Surveillance Epidemiology and End Results (SEER) program, which maintains a nationally representative sample of cancer patients (7–12). HPV-related OPSCC are typically estimated based on anatomic sites, of which about 30% are not actually HPV-related (7, 13). Mahal et al. estimated the incidence and demographics of HPV-associated OPSCC patients using SEER data with HPV status information, although almost half of these patients lacked information on HPV status (13). The SEER data only cover about one-fourth of the US population. In contrast, United States Cancer Statistics (USCS) gathers information on cancer cases and patient demographics for essentially the entire US population, which provides an opportunity to accurately estimate cancer incidence trends in the entire US population. Furthermore, close examination of the trends in incidence of HPV-related OPSCC across age groups, race/ethnic groups, and regions of residence among both male and female adults in the US is lacking. The HPV vaccine was approved in 2006 for females and in 2009 for males, and was shown to decrease vaccine-related HPV types in the oropharynx (14). Currently, the Centers for Disease Control and Prevention (CDC)’s Advisory Committee on Immunization Practices (ACIP) recommends a two-dose HPV immunization schedule for persons who initiate the vaccine series at ages 9 through 14 years, while a three-dose schedule is recommended for those 15-26 years of age (15). For adults 27-45 years of age, the ACIP recommends them to get medical counseling about their risk for new HPV infections and potential benefits of vaccination (16). In the US, almost all eligible persons have coverage for HPV vaccine from multiple sources of private and public financing. The impact of HPV vaccine on the incidence of oropharyngeal cancers has not been examined. We investigated the impact of HPV vaccination on the incidence of HPV-related OPSCC in the US among male and female adults from different age groups.

## Methods

We used the USCS database 2001–2018, which combines data from the Centers for Disease Control and Prevention (CDC)’s National Program for Cancer Registries (NPCR) and the SEER program (17). Both contain data on patient demographics and tumor characteristics from hospitals, physicians, and laboratories across the nation. The USCS database 2001–2018 represents the entire US population (excluding Puerto Rico) between 2001 and 2018. The Institutional Review Board at The University of Texas Medical Branch did not consider this study human subjects research; therefore, it did not require approval.

We classified OPSCC cases potentially due to HPV based on anatomic sites identified by ICD-0-3 as follows: base of tongue (C01.9), lingual tonsil (C02.4), overlapping lesion of tongue (C02.8), soft palate (C05.1), uvula (C05.2), tonsil (C09.0-C09.1, C09.8-C09.9), vallecula (C10.0), anterior surface of epiglottis (C10.1), oropharyngeal wall (C10.2-10.3), branchial cleft (C10.4), pharynx (C14.0), Waldeyer ring (C14.2), or other oropharynx site (C10.8-C10.9, C14.8). Invasive squamous cell cases were defined using ICD-O-3 histology codes 8050-8086 and 8120-8131 and only microscopically confirmed cases were included (8). Each case had patient demographics and the cancer diagnosis date. We stratified the data by age and region of residence - Northeast, Midwest, South, and West. Analyses included information about race and ethnicity. Race was grouped into Non-Hispanic White, Non-Hispanic Black, Asian/Pacific Islander, and Other categories, and ethnicity was classified as either Hispanic or non-Hispanic. The North American Association of Central Cancer Registries (NAACCR) Hispanic/Latino Identification Algorithm (NHIA) was used to identify Hispanic ethnicity for all cancer cases (18).

### Statistical Analysis

The SEER*Stat statistical software package (version 8.3.8) and SAS for Windows version 9.4 (SAS Institute) were used to conduct the analyses. Differences with two-tailed P values < 0.05 were considered statistically significant. OPSCC incidence rates were calculated as the number of cases per 100,000 persons and were age-adjusted to the 2000 US standard population. The Tiwari method was used to determine the confidence intervals (CI) (19). Annual percentage changes (APCs) in incidence were calculated using the equation, (exp[β]-1)*100. A least-squares regression line was fitted to the natural logarithm of the rates, using the calendar year as a regressor variable, to estimate the regression coefficient (β). The statistical significance of APCs and differences between APCs were determined using tests based on previously proposed methods (20). Joinpoint regression uses least squares regression to fit line segments to the natural log of the age-standardized incidence rates, joined at discrete points that represent statistically significant changes in the direction of the trend. Joinpoint regression was performed using the Joinpoint Regression Program from National Cancer Institute. The 5–year average annual incidence rates were calculated for 5 years before the introduction of HPV vaccination (2002–2006) and the latest 5 years in the vaccine era (2014–2018). Differences in age–adjusted rates were evaluated using rate ratios (RRs) and the corresponding 95% confidence intervals (CIs).

Cancer-specific five-year survival probability was calculated using 60 monthly intervals in SEER*Stat software. Data used were from SEER’s 18 registry areas. SEER*Stat software used expected life tables instead of a cohort of cancer-free individuals, assuming that the cancer deaths were a negligible proportion of all deaths. Individuals who died of causes other than OPSCC were considered censored when we estimated cancer-specific survival. Cox proportional hazard models were fitted to compare differences in 5-year survival probability across time by stage at diagnosis, controlling for age at diagnosis and race/ethnicity. Hazard ratios (HRs) and 95% CIs were estimated from the Cox model. Kaplan-Meier curves were plotted to show differences in cumulative probability of death across time. We had successfully used the same software package and similar methods to examine incidence of cervical cancer and breast cancer, and cancer-specific survival (21–23).

## Results

There were 229,264 adult males and 55,108 adult females diagnosed with HPV-related oropharyngeal squamous cell carcinoma (OPSCC) from 2001 to 2018. Among these male patients, 4.6% were 20-44 years old, 61.7% were 45-64 years old, and 33.7% were 65+ years old, while among these female patients 5.4% were 20-44 years old, 50.8% were 45-64 years old, and 43.8% were 65+ years old (**Supplemental Table 1**).

**Figure 1:**
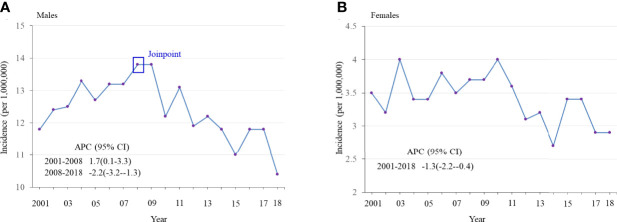
Age-adjusted incidence of HPV-related oropharyngeal squamous cell carcinoma (OPSCC) from 2001 to 2018 among adults 20-44 years old, stratified by sex. **(A)** Males **(B)** Females.

Age-adjusted annual incidence of OPSCC was significantly lower in 2014-2018 than in 2002-2006 among males 20-44 years old (11.4 vs 12.8 per 1,000,000, rate ratio 0.89, 95% confidence interval 0.84-0.93, **Table 1**) and among females (3.0 vs 3.6 per 1,000,000, rate ratio 0.86, 95% confidence interval 0.78-0.95), but increased among those 45-64 years old (220.3 vs 170.8 per 100,000 in males, rate ratio 1.29, 95% confidence interval 1.27-1.31; 39.1 vs 33.9 per 100,000 in females, rate ratio 1.15, 95% confidence interval 1.12-1.19) and those 65+ years old (284.4 vs 174.6 per 100,000 in males, rate ratio 1.63, 95% confidence interval 1.60-1.66; 59.0 vs 55.1 per 100,000 in females, rate ratio 1.07, 95% confidence interval 1.04-1.11). Overall negative trends of OPSCC incidence were observed for adults 20-44 years old of both sexes and joinpoint regression revealed a significant joint in the HPV-OPSCC incidence trend for 20-44-year-old males in 2008, after which incidence began to decrease (**Figure 1**). No joints were observed among females of the same age group. Trends in HPV-related OPSCC incidence among males and females 45-64 years old and 65+ years old generally increased over time (**Supplemental Figures 1, 2**). Independent of age group, OPSCC incidence was consistently and significantly greater among male patients.

**Table 1:** Age-adjusted incidence of HPV-related oropharyngeal squamous cell carcinoma (OPSCC) among US adults by age group and sex during 2002-2006 and 2014-2018.

	Incidence (per 1,000,000 person-years)		Rate ratio 2014-2018/2002-2006
	2002-2006	2014-2018	RR (95% CI)
Male			
20-44 years old			
All	12.8 (12.4-13.3)	11.4 (10.9-11.8)	0.86 (0.78-0.95)
**Race/Ethnicity**			
Hispanic	5.4 (4.7-6.2)	5.0 (4.4-5.7)	0.93 (0.77-1.14)
Non-Hispanic White	14.8 (14.3-15.4)	14.6 (13.9-15.3)	0.98 (0.93-1.04)
Non-Hispanic Black	12.8 (11.5-14.1)	10.1 (9.0-11.3)	0.79 (0.67-0.92)
Asian/Pacific Islander	4.2 (3.1-5.5)	3.5 (2.7-4.6)	0.85 (0.57-1.26)
Region			
Northeast	11.2 (10.2-12.2)	3.4 (2.9-3.9)	0.95 (0.83-1.08)
Midwest	13.4 (12.5-14.4)	3.5 (3.0-4.0)	1.02 (0.92-1.14)
South	15.5 (14.7-16.3)	4.3 (3.9-4.8)	0.81 (0.74-0.87)
West	9.5 (8.7-10.3)	2.6 (2.2-3.0)	0.87 (0.76-0.98)
45-64 years old			
All	170.8 (168.8-172.7)	220.3 (218.3-222.3)	1.15 (1.12-1.19)
**Race/Ethnicity**			
Hispanic	92.6 (87.7-97.7)	92.8 (89.2-96.5)	1.00 (0.94-1.07)
Non-Hispanic White	182.7 (180.4-185.0)	266.6 (263.9-269.2)	1.46 (1.44-1.48)
Non-Hispanic Black	198.8 (192.2-205.6)	152.7 (147.8-157.6)	0.77 (0.73-0.80)
Asian/Pacific Islander	39.0 (34.4-43.9)	47.8 (43.8-52.1)	1.23 (1.06-1.43)
Region			
Northeast	161.6 (157.3-166.0)	33.0 (31.2-35.0)	1.29 (1.25-1.34)
Midwest	164.6 (160.6-168.6)	34.6 (32.8-36.4)	1.43 (1.38-1.47)
South	193.1 (189.7-196.6)	37.7 (36.3-39.2)	1.27 (1.24-1.29)
West	149.2 (145.3-153.1)	27.7 (26.1-29.4)	1.19 (1.15-1.23)
65+ years old			
All	174.6 (171.7-177.6)	284.4 (281.2-287.6)	1.07 (1.04-1.11)
**Race/Ethnicity**			
Hispanic	149.4 (138.1-161.4)	187.4 (178.2-197.1)	1.25 (1.14-1.38)
Non-Hispanic White	175.7 (172.5-179.0)	310.7 (307.0-314.4)	1.77 (1.73-1.81)
Non-Hispanic Black	223.1 (211.2-235.5)	227.2 (217.3-237.5)	1.02 (0.95-1.09)
Asian/Pacific Islander	72.7 (61.9-84.8)	83.0 (75.0-91.7)	1.14 (0.95-1.38)
Region			
Northeast	170.8 (164.3-177.5)	53.5 (50.4-56.7)	1.56 (1.49-1.64)
Midwest	159.6 (153.8-165.6)	53.9 (51.0-57.0)	1.69 (1.62-1.77)
South	192.7 (187.6-197.9)	56.3 (53.9-58.7)	1.60 (1.55-1.65)
West	163.5 (157.4-169.9)	56.0 (52.8-59.3)	1.66 (1.58-1.73)
Female			
20-44 years old			
All	3.6 (3.3-3.8)	3.0 (2.8-3.3)	0.89 (0.84-0.93)
**Race/Ethnicity**			
Hispanic	1.7 (1.3-2.2)	1.9 (1.5-2.3)	1.14 (0.81-1.61)
Non-Hispanic White	4.0 (3.7-4.3)	3.8 (3.4-4.1)	0.95 (0.85-1.07)
Non-Hispanic Black	4.0 (3.3-4.7)	2.4 (1.9-3.0)	0.60 (0.45-0.80)
Asian/Pacific Islander	2.2 (1.5-3.2)	1.5 (1.0-2.1)	0.66 (0.38-1.14)
Region			
Northeast	10.6 (9.6-11.7)	3.3 (2.7-3.9)	0.96 (0.76-1.22)
Midwest	13.7 (12.6-14.8)	3.5 (3.0-4.0)	1.00 (0.81-1.23)
South	12.5 (11.8-13.3)	3.4 (3.0-3.8)	0.79 (0.68-0.92)
West	8.2 (7.5-9.0)	2.0 (1.6-2.4)	0.77 (0.60-0.99)
45-64 years old			
All	33.9 (33.1-34.7)	39.1 (38.3-39.9)	1.29 (1.27-1.31)
**Race/Ethnicity**			
Hispanic	14.7 (12.8-16.7)	18.0 (16.5-19.7)	1.23 (1.05-1.44)
Non-Hispanic White	36.4 (35.4-37.4)	46.2 (45.1-47.3)	1.27 (1.23-1.32)
Non-Hispanic Black	41.0 (38.3-43.8)	33.9 (31.8-36.1)	0.83 (0.75-0.91)
Asian/Pacific Islander	9.1 (7.1-11.5)	10.1 (8.4-11.9)	1.11 (0.82-1.50)
Region			
Northeast	208.5 (204.0-213.1)	37.9 (36.0-39.8)	1.15 (1.06-1.24)
Midwest	234.7 (230.3-239.1)	41.2 (39.4-43.1)	1.19 (1.11-1.28)
South	244.4 (241.0-247.8)	43.8 (42.4-45.2)	1.16 (1.10-1.22)
West	177.2 (173.5-180.9)	30.2 (28.7-31.8)	1.09 (1.01-1.18)
65+ years old			
All	55.1 (53.6-56.5)	59.0 (57.7-60.4)	1.63 (1.60-1.66)
**Race/Ethnicity**			
Hispanic	31.5 (27.2-36.3)	33.5 (30.2-37.1)	1.06 (0.89-1.27)
Non-Hispanic White	58.7 (57.1-60.4)	66.3 (64.7-67.9)	1.13 (1.09-1.17)
Non-Hispanic Black	50.1 (45.6-54.8)	39.7 (36.4-43.3)	0.79 (0.70-0.90)
Asian/Pacific Islander	20.4 (15.7-26.2)	23.0 (19.3-27.2)	1.13 (0.83-1.55)
Region			
Northeast	267.0 (259.9-274.4)	61.5 (58.5-64.7)	1.15 (1.06-1.24)
Midwest	270.1 (263.5-276.8)	59.9 (57.1-62.8)	1.11 (1.03-1.20)
South	308.7 (303.4-314.1)	61.4 (59.3-63.6)	1.09 (1.03-1.15)
West	271.0 (264.5-277.6)	52.0 (49.4-54.7)	0.93 (0.86-1.00)

**Figure 2:**
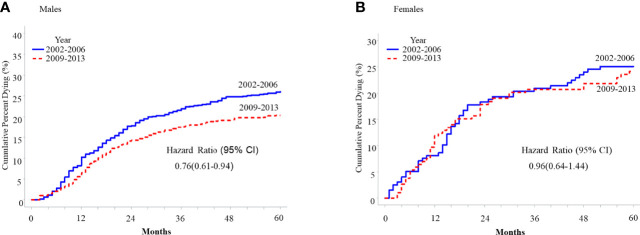
Five-year cumulative probability of death from HPV-related oropharyngeal squamous cell carcinoma (OPSCC) among cancer patients 20-44 years old with HPV-related OPSCC, SEER 2001-2018, stratified by sex. **(A)** Males **(B)** Females.

Cancer-specific 5-year survival was significantly improved across age groups among both males and females (**Table 2**), except for 20-44-year-old females (74.8% in 2002-2006 vs. 75.7% in 2009-2013, hazard ratio 0.96, 95% CI 0.64-1.44, p=0.84, **Figure 2**). Hazard ratios obtained from Kaplan-Meier curves of the cumulative probability of death from OPSCC across time among male or female patients 45-64 years old and 65+ years old were lower among men for both age groups relative to women (**Supplemental Figures 3, 4**).

**Table 2:** Cancer-specific five-year survival among adult males and females with HPV-related oropharyngeal squamous cell carcinoma (OPSCC), SEER 2002-2006 and 2009-2013 (N=29304).

	5-year survival % (95% CI)	
	2002-2006	2014-2018
Male		
20-44 years old		
All	73.8 (70.5-76.9)	79.6 (76.3-82.4)
**Race/Ethnicity**		
Hispanic	71.3 (57.9-81.1)	86.6 (76.4-92.5)
Non-Hispanic White	78.1 (74.4-81.3)	81.6 (77.9-84.8)
Non-Hispanic Black	47.3 (35.9-57.9)	56.7 (44.4-67.3)
Asian/Pacific Islander	73.7 (47.9-88.1)	76.8 (57.3-88.2)
45-64 years old		
All	69.2 (68-70.3)	75.7 (74.8-76.6)
**Race/Ethnicity**		
Hispanic	67.3 (62.5-71.7)	69.8 (65.9-73.3)
Non-Hispanic White	73.4 (72.1-74.5)	78.4 (77.4-79.3)
Non-Hispanic Black	40.7 (37.1-44.2)	55.7 (52.2-59.0)
Asian/Pacific Islander	68.9 (60.3-76.0)	75.9 (68.9-81.5)
65+ years old		
All	51.3 (49.2-53.3)	62.9 (61.3-64.5)
**Race/Ethnicity**		
Hispanic	39.9 (31.7-48.0)	53.7 (47.2-59.7)
Non-Hispanic White	54.7 (52.4-57.0)	65.2 (63.4-66.9)
Non-Hispanic Black	30.9 (25.0-37.0)	44.4 (38.3-50.3)
Asian/Pacific Islander	52.4 (41.1-62.6)	64.5 (54.9-72.6)
Female		
20-44 years old		
All	74.8 (68.1-80.3)	75.7 (68.8-81.3)
**Race/Ethnicity**		
Hispanic	80.0 (50.0-93.1)	67.6 (38.3-85.2)
Non-Hispanic White	77.0 (68.7-83.3)	80.3 (72.4-86.1)
Non-Hispanic Black	50.4 (32.3-66.0)	40.6 (19.5-60.9)
Asian/Pacific Islander	100	92.3 (56.6-98.9)
45-64 years old		
All	67 (64.3-69.6)	71.8 (69.5-73.9)
**Race/Ethnicity**		
Hispanic	66.6 (53.7-76.6)	73.6 (64.7-80.5)
Non-Hispanic White	70.1 (67.1-73.0)	75.6 (73.1-78.0)
Non-Hispanic Black	47.9 (40.3-55.1)	47.3 (40.3-54.0)
Asian/Pacific Islander	88.5 (68.4-96.1)	80.4 (65.6-89.3)
65+ years old		
All	50.7 (47.4-53.9)	57.6 (54.5-60.6)
**Race/Ethnicity**		
Hispanic	53.1 (38.1-66.0)	63.5 (49.9-74.3)
Non-Hispanic White	51.4 (47.7-54.9)	58.9 (55.4-62.2)
Non-Hispanic Black	45.3 (33.4-56.4)	49.1 (38.9-58.5)
Asian/Pacific Islander	33.4 (15.9-52.0)	45.0 (30.8-58.2)

## Discussion

This study demonstrated a decline in the incidence of HPV-related OPSCC among young males and females during the vaccination era (2014–2018) compared with the pre-vaccination era (2002–2006). Previous research established that changes in HPV prevalence are the primary driver behind increased OPSCC incidence (7, 9, 10). It is likely that HPV vaccination of young girls and boys and the resulting decreased oral HPV infection rates, as well as emerging herd immunity (starting after the 2006 approval of the HPV vaccine in the US), may be partially responsible for this observed decrease in OPSCC incidence among young adults (14, 24). Additionally, smoking rates have been declining since the 1980s, especially among young adults. This decline may also contribute to the decreased incidence in this age group as tobacco use increases susceptibility to oral HPV infection (25, 26). Although HPV vaccine uptake rates steadily increased from 2008 to 2016 (53.6% vs. 65.1%), the Healthy People 2020 goal of vaccinating 80% of all teenagers 13-15 years old has not been met (27). Additional efforts are needed to improve HPV vaccination coverage among young girls and boys to reduce the future burden of HPV-related OPSCC.

Previous literature showed increasing trends in the incidence of HPV-related OPSCC among middle-aged and elder adults in the US (7–12). We also observed an increased incidence of HPV-related OPSCC in middle-aged and elder males and females, which is consistent with those findings (7–12). Data from SEER indicate that increasing trends in HPV-related OPSCC were primarily observed in middle-aged individuals and elders, particularly within recent years (7–9). In contrast, an international study by Chaturvedi et al. that examined the incidence of OPSCC using data from the Cancer Incidence in Five Continents database found increasing incidence in 7 out of 9 counties examined between the years of 1983 to 2002, with significantly stronger increases among patients <60 years old compared to patients ≥ 60 years old (10). Previous research has widely established that changes in HPV prevalence are the primary driver behind increased OPSCC incidence (3, 7–10, 28). High oral HPV infection rates, especially among males, for these age groups can partially explain the observed increase (29, 30). Trends in smoking may also be partially responsible for changes in HPV-related OPSCC incidence (25, 31).

Overall, our data show that HPV-related OPSCC was much more common in males. This likely reflects the higher oral HPV infection rates observed among men (29, 30). Among younger males, a significant inflection point in the trends of OSPCC incidence occurred in 2008, after which the incidence of OPSCC began to decrease. This did not occur among female adults. Among young females, the incidence of OPSCC decreased slightly but gradually during 2001-2018, which may be partly due to HPV vaccination, low prevalence of oral HPV infection (29, 30), and rising use of tobacco. Five-year survival rates also significantly improved among younger males, but not younger females, which may be due to hesitance to administer aggressive multimodality therapy and differences in utilization of definitive treatments. This may also explain why hazard ratios were significantly lower and survival was significantly improved among men in older age groups as well.

The findings of our study have important health implications. Our observed increasing trends in HPV-related OPSCC among middle-aged and elder males and females call for interventions to reduce risk factors for this type of cancer. Notable actionable risk factors include oral HPV infection (29, 30) and smoking (25, 26, 31). Therefore, avoiding high-risk sex behaviors and tobacco control remain the primary prevention methods to alleviate the burden of HPV-related OPSCC among the US adult population. Our observed sex-based disparities in OPSCC incidence and 5-year cancer survival rates also necessitate future mitigative actions. Such disparities could be reduced by taking measures to mitigate the rising tobacco use among women. Most encouragingly, the potential linkage between decreasing trends in the incidence of HPV-related OPSCC among young adults especially after the introduction of HPV vaccination in 2006 and the high HPV vaccination uptake among young girls and boys in the US indicates the effectiveness of the HPV vaccine.

One strength of our study is the quality of the data from the USCS database, itself comprised from both NPCR and SEER data. In addition to the NPCR/SEER data that cover a diverse cross-section of the entire US population, the USCS includes patient sociodemographics, cancer diagnosis date, and age. Nevertheless, our study has a few limitations. Since HPV infection status is not available for participants in USCS, we classified potentially HPV-related OPSCC based on anatomic site, rather than through direct assessment of HPV DNA-positivity (e.g., *via* p16 immunohistochemistry). Modern estimates of HPV DNA-positive tumor sites classified as HPV-related based on anatomic site are merely 70%; therefore, these anatomic site-based classifications may have led to the occasional misclassification (7). Based on 2003-2004 SEER data from 3917 OPSCC patients with known HPV status, 2903 (74.1%) were HPV positive (13). The data used in this study also lacked information on other risk factors for HPV-related OPSCC. Certain subgroup analyses, such as for sex, racial, and age groups, could not be conducted due to an insufficient number of cases of OPSCC in females. Our study also has the following limitations: potential misclassification bias, residual confounders, potential period effect, and birth cohort effect of the US population.

## Conclusion

HPV-related OPSCC was much more common in males and is likely attributable to the higher oral HPV infection rates previously observed among men (29, 30). HPV-related OPSCC incidence declined among young adult (20–44) males and females during the vaccination era compared to the pre-vaccination era, suggesting that HPV vaccinations are beginning to reduce OPSCC burden. In contrast, the incidence of HPV-related OPSCC increased among vaccine-ineligible middle-aged (45–64) and elder (65+) males and females. Cancer-specific 5-year survival was significantly improved in young males but not in young females. Additional efforts are needed to improve HPV vaccination coverage in young girls and boys to further reduce the burden of HPV-related OPSCC in the US.

## Data Availability Statement

Publicly available datasets were analyzed in this study. This data can be found here: the National Program for Cancer Registries (NPCR) and the Surveillance, Epidemiology, and End Results (SEER) databases (2001-2018).

## Ethics Statement

This study did not involve human subjects and did not require approval by the Institutional Review Board of The University of Texas Medical Branch.

## Author Contributions

Study concept, methodology, data analyses, manuscript preparation, and funding (FG). Data analyses and manuscript preparation (MC). Manuscript preparation (MS). Study concept and manuscript preparation (BK). Study concept, methodology, data analysis, manuscript preparation, and funding (AB). All authors contributed to the article and approved the submitted version.

## Funding

Dr. Guo is currently supported by an award (K07CA222343) from the National Institutes of Health, National Cancer Institute (NIH/NCI). This study was supported by an award to Dr. Berenson (PP200005) from the Cancer Prevention and Research Institute of Texas (CPRIT). The content is solely the responsibility of the authors and does not represent the official views of the NIH/NCI or CPRIT.

## Conflict of Interest

The authors declare that the research was conducted in the absence of any commercial or financial relationships that could be construed as a potential conflict of interest.

## Publisher’s Note

All claims expressed in this article are solely those of the authors and do not necessarily represent those of their affiliated organizations, or those of the publisher, the editors and the reviewers. Any product that may be evaluated in this article, or claim that may be made by its manufacturer, is not guaranteed or endorsed by the publisher.
