# Supplementary material for: Trends in Oropharyngeal Cancer Incidence Among Adult Men and Women in the United States From 2001 to 2018
Source: Front Oncol. 2022 Jul 18;12:926555. doi: 10.3389/fonc.2022.926555 (PMC9339703; doi:10.3389/fonc.2022.926555)

Supplemental Table 1. Basic characteristics of US adult males and females with HPV-related oropharyngeal squamous cell carcinoma (OPSCC) from 2001 to 2018 (N=284,372)

**Characteristics**	**Frequency (%)**	
	**Male**	**Female**
**Age**		
20-44	10,594(4.6)	2,981(5.4)
45-64	141,470(61.7)	27,973(50.8)
65+	77,200(33.7)	24,154(43.8)
**Race**		
Hispanic	11,361(5.0)	2,533(4.6)
Non-Hispanic White	192,494(84.0)	45,606(82.8)
Non-Hispanic Black	20,565(9.0)	5,612(10.2)
Asian/Pacific Islander	2,555(1.1)	835(1.5)
**Region**		
Northeast	40,164(17.5)	10,489(19.0)
Midwest	50,503(22.0)	12,353(22.4)
South	93,829(40.9)	22,115(40.1)
West	44,768(19.5)	10,151(18.4)

Supplemental Figure 1. Age-adjusted incidence of HPV-related oropharyngeal squamous cell carcinoma (OPSCC) from 2001 to 2018 among adults 45-64 years old, stratified by sex. A: Males B: Females

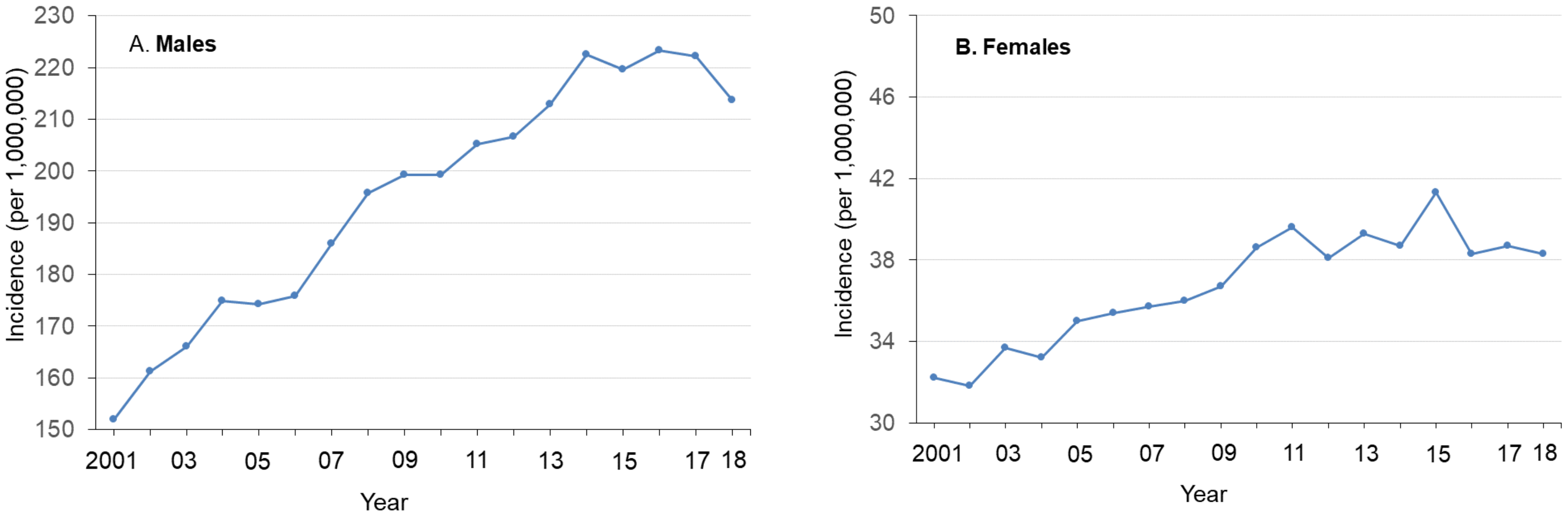

Supplemental Figure 2. Age-adjusted incidence of HPV-related oropharyngeal squamous cell carcinoma (OPSCC) from 2001 to 2018 among adults 65+ years old, stratified by sex. A: Males B: Females

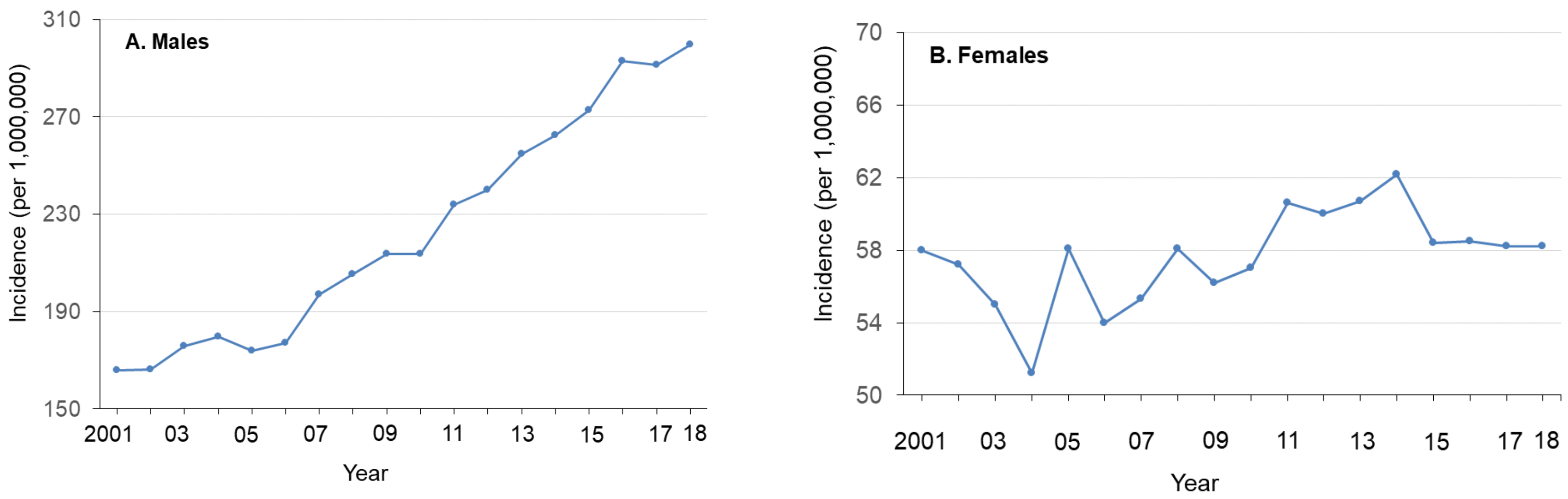

Supplemental Figure 3. Five-year cumulative probability of death from HPV-related oropharyngeal squamous cell carcinoma (OPSCC) among cancer patients 45-64 years old with HPV-related OPSCC, SEER 2002-2006 and 2009-2013, stratified by sex. A: Males B: Females

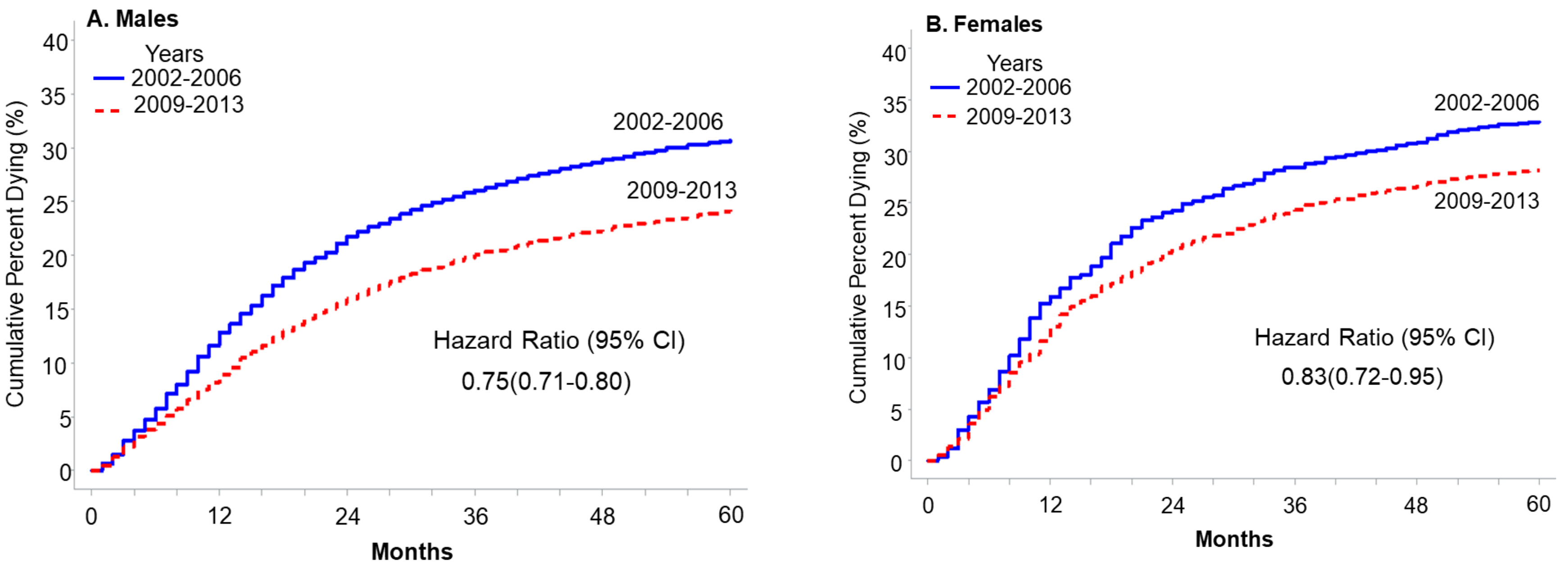

Supplemental Figure 4. Five-year cumulative probability of death from HPV-related oropharyngeal squamous cell carcinoma (OPSCC) among cancer patients 65+ years old with HPV-related OPSCC, SEER 2002-2006 and 2009-2013, stratified by sex. A: Males B: Females

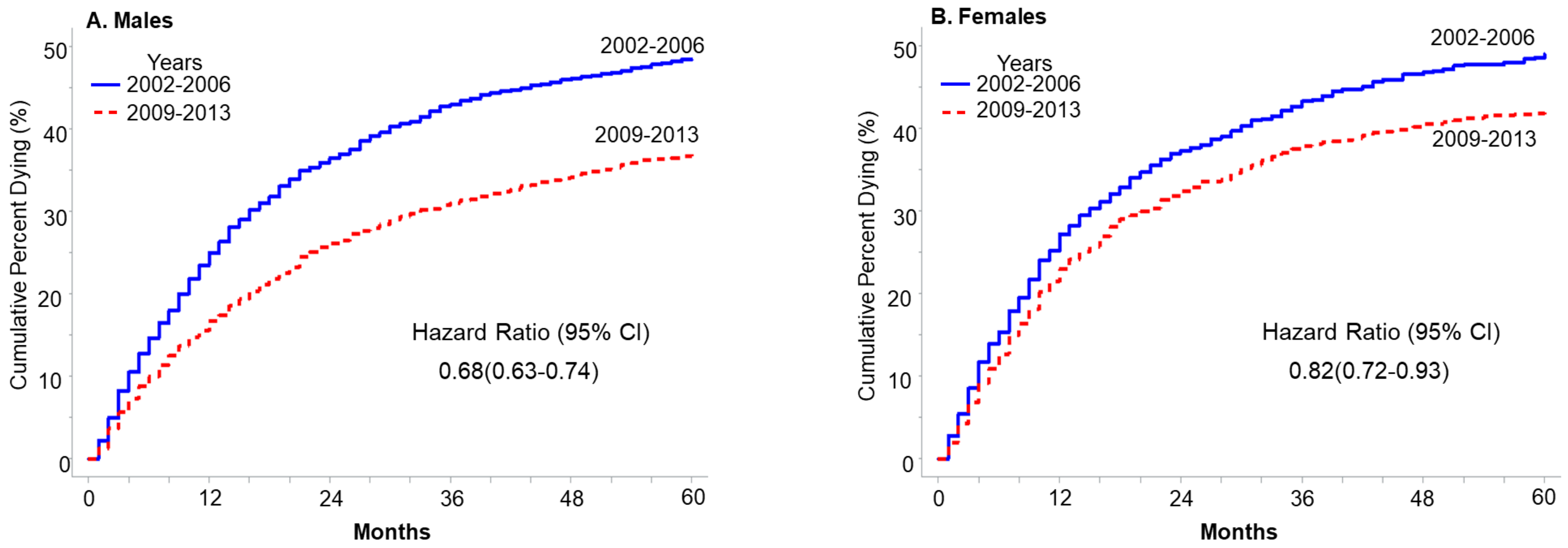

Supplement: Supplementary file 1 [file DataSheet_1.pdf]
